# Inspection on the Mechanism of SARS-CoV-2 Inhibition by Penciclovir: A Molecular Dynamic Study

**DOI:** 10.3390/molecules28010191

**Published:** 2022-12-26

**Authors:** Micaela Giannetti, Claudia Mazzuca, Giorgio Ripani, Antonio Palleschi

**Affiliations:** Department of Chemical Science and Technologies, University of Rome “Tor Vergata”, Via della Ricerca Scientifica, 00133 Rome, Italy

**Keywords:** SARS-CoV-2, Penciclovir, nsp12, RNA, molecular dynamic simulations, drug, Gromacs19, nucleotide analog

## Abstract

In recent years, humanity has had to face a critical pandemic due to SARS-CoV-2. In the rapid search for effective drugs against this RNA-positive virus, the repurposing of already existing nucleotide/nucleoside analogs able to stop RNA replication by inhibiting the RNA-dependent RNA polymerase enzyme has been evaluated. In this process, a valid contribution has been the use of in silico experiments, which allow for a rapid evaluation of the possible effectiveness of the proposed drugs. Here we propose a molecular dynamic study to provide insight into the inhibition mechanism of Penciclovir, a nucleotide analog on the RNA-dependent RNA polymerase enzyme. Besides the presented results, in this article, for the first time, molecular dynamic simulations have been performed considering not only the RNA-dependent RNA polymerase protein, but also its cofactors (fundamental for RNA replication) and double-strand RNA.

## 1. Introduction

During recent years, severe infective diseases have been caused by RNA-positive viruses, including acute hepatitis A, chronic hepatitis C, hemorrhagic fevers, meningitis, encephalitis, paralytic poliomyelitis, Ebola virus (EBV), severe acute respiratory syndrome coronavirus (SARS-CoV), and the more recent severe acute respiratory syndrome coronavirus 2 (SARS-CoV-2). Like Ebola virus, coronavirus has already been characterized and well-studied since 2003. Like every RNA virus, both SARS-CoV and SARS-CoV-2 use, to replicate their genetic material the RNA-dependent RNA polymerase (RdRp), which therefore plays a key role in the replication and transcription processes during the life of the RNA virus.

The RNA-dependent RNA polymerase (RdRp) derives from a common gene encoded by the RNA virus [1], and the RdRp protein plays central roles in the DNA-independent genome replication and transcription processes essential for the RNA virus life cycle. The process of RNA elongation through RdRp catalysis is well described by X-ray crystal data and Cryo-EM [2,3,4] and MD simulation [5,6], elucidating the key features in the RdRp catalytic mechanism of synthesis and RNA translocation.

Although viral RdRps are different in size and structural organization, all of them share a well-conserved sequence motif (about 500 residues) of the core structure [3], divided into three domains [1]: fingers, palm, and thumb. In turn, they contain the seven most conserved motifs (called A-G) arranged around the catalytic site, encircling it and conferring to the protein a right-hand-like shape. In this way, the structure assumes a closed-hand feature and forms two positively charged channels in which are located the template and the nucleotide three phosphate (NTP) binding site.

In the case of SARS-CoV-2, its replication is performed by the RdRp C terminal domain (residues 398-815) of the non-structural protein nsp12.

The whole replicase function is exerted by not only the RNA polymerase (nsp12, as already explained), but also a helicase (nsp13) and rare or even unique domains involved in mRNA capping (nsp14, nsp16) and fidelity control (nsp14). Several smaller subunits (nsp7-nsp10) act as crucial cofactors of these enzymes [7], as reported in detail in SM.

A multi-step mechanism for the cyclic addition of nucleotides in the RNA replication of the RdRp has been proposed [2] based on the poliovirus polymerase elongation complexes. First, the catalytic site is opened and empty, allowing a nucleotide triphosphate (NTP) to enter inside the active site of the enzyme. This, in turn, promotes a rearrangement of the catalytic residues and of the two metal ions approaching the NTP to allow the addition reaction of the nucleotide to the RNA product strand. Finally, the translocation of the dsRNA, leaving a new position free for the entering of next NTP, occurs. The product–template hybrid leaves the active site through the RNA exit tunnel at the front side of the polymerase [4], thus enabling another step in the nucleotide elongation cycle’s process. It should be noted that the catalytic mechanism is mediated by two charged bivalent cations (M^2+^) inside the active site [8,9]. One of the two cations (called, in the following, MB) lowers the pKa value of O3′ of the ribose ring of the last nucleotide of the product strand, while a second cation (called, in the following, MA) stabilizes the penta-coordinate transition state and helps the exit of the leaving pyrophosphate group [10].

The knowledge of the RNA replication mechanisms in RdRp is of primary importance in looking for new, highly efficient drugs such as nucleotide/nucleoside analogs (NAs), an important class of small molecule compounds targeting various types of polymerases, and allows a rapid investigation, experimentally or in silico, about the capability of the repurposing of antiviral, antibacterial, or antifungal drugs. In more detail, in cases in which no therapeutic agents are rapidly available, as occurred in 2019 during the COVID-19 pandemic, already existing drugs have been evaluated against the new therapeutic target, which, in the case of SARS-CoV-2, is the RdRp nsp12 protein. This is the case, for example, of Remdesivir, Sofosbuvir, Tenofovir, and Ribavirin [11,12,13].

The reuse of existing small molecule antiviral drugs is, indeed, an important and promising strategy for addressing the COVID-19 epidemic. If the existing drugs can be repurposed for the treatment of SARS-CoV-2 infection, preclinical research (such as animal experiments and pharmacological research) and early clinical research can be bypassed, and the drugs can directly enter phase II or III clinical trials [14].

In this context, for example, molecular docking allows for the evaluation of binding energies and the mode of physicochemical interaction of the active site of the protein with the drugs [15,16] in a relatively rapid and low-cost way; on the basis of docking scores and binding energy thus obtained, it is possible to estimate the more promising therapeutic agents. However, this procedure has several drawbacks. Docking calculations, indeed, do not consider the solvent explicitly (therefore underestimating the hydrophobic effect) and do not take into account internal motions of the ligand, nor the internal degree of freedom of the receptor [17,18]. Dynamics effects and explicit solvent treatment are essential features that can be reached, in silico, via molecular dynamics only. Moreover, also in the case in which molecular simulations were performed, cofactors and/or the presence of the double-strand RNA (dsRNA; template and product) inside the binding pocket are discarded even if they are essential features for the replication mechanism [19,20,21,22,23,24]. The role of the cofactors is indeed to help the proper replication mechanism, stabilizing the dsRNA and thus maintaining fidelity of genome replication. It is noteworthy, in this context, the studies performed by Zhang and coworkers [19]. They, using the computational technique of molecular docking, performed on three viral RdRp inhibitors (Galidesivir, Favipiravir, and Penciclovir) which were reported to have clinical significance for SARS-CoV-2 [23,24] on nsp12, and they obtained a structural model for each ligand–receptor pair. Results are compared with a positive control, i.e., a docking calculation with one of the four natural nucleotides of the RNA: adenosine triphosphate. In their calculations, metal ions are absent, even if they are essential for the replication mechanism.

**Scheme 1:**
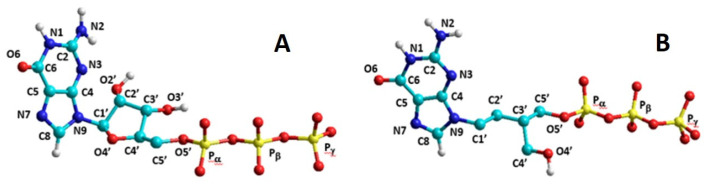
Structures of (**A**): Guanosine triphosphate (GTP) and (**B**): Penciclovir triphosphate (PENT). Oxygens are depicted in red, nitrogens in blue, phosphates in yellow, carbons in cyan, and hydrogens in white colors.

In this work we present molecular dynamics (MD) simulations of a potential drug against SARS-CoV-2, that is, Penciclovir triphosphate (PENT, Scheme 1), during some steps (see above) of the RNA elongation process, comparing the results with those obtained by using the base guanosine triphosphate (GTP, Scheme 1). PENT, a guanosine triphosphate analog, is one of the potential anti-SARS-CoV-2 drugs; in more detail, this drug is active against infection of the herpes simplex virus, and several in silico and in vitro studies have proposed it for repurposing against SARS-CoV-2 nsp12 [16]. It is part of a larger class of RdRp inhibitors, namely nucleoside inhibitors (Nis), that terminate the RNA synthesis step, which, in turn, is essential for RNA replication. One possibility explaining their function is the steric hindrance of NIs, carrying an alcohol group in the C3′ instead of the heterocyclic moiety as proposed for Remdesivir [25]. Due to this mechanism, NIs are sometimes called chain termination inhibitors [26].

**Figure 1:**
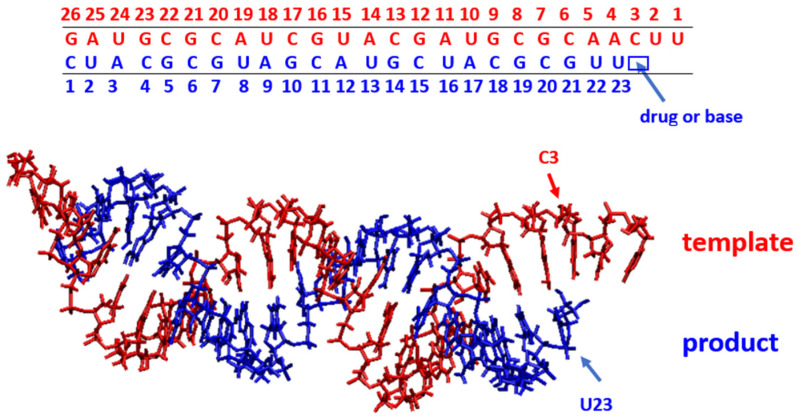
Sequence (top) and structure (bottom) of the dsRNA, template (red) and product (blue), used in the MD simulations of the complex with unbound and bound (in +1 position) drug or base.

**Figure 2:**
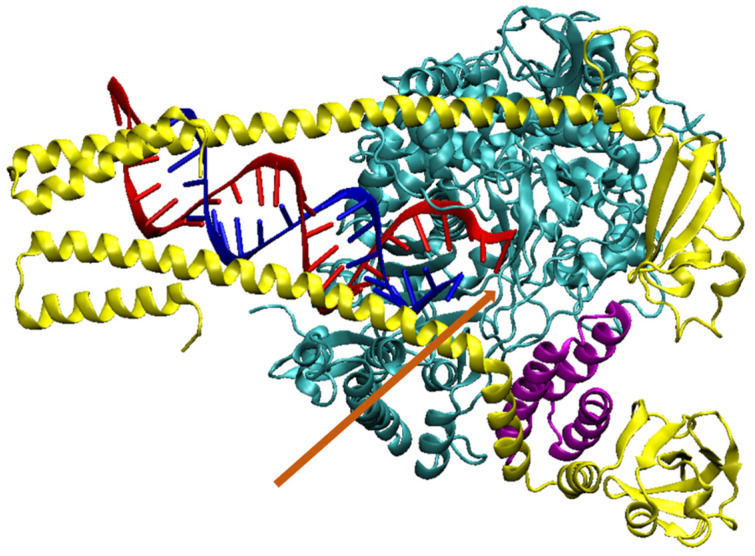
Structure of the SARS-CoV-2 replication complex. nsp12 (cyan), nsp7 (magenta), nsp8a, b (yellow), product strand (blue), and template strand (red). The VMD software is used to obtain this image. The orange arrow indicates the active site.

This article is to obtain a twofold purpose. The first is the design of molecular dynamics simulations that, for the first time, to the best of our knowledge, include a stable ds-RNA in which template and product strands are present (Figure 1 and Figure 2).

Moreover, the other novelty with respect to the literature is that in the MD simulations, not only are nsp7 and nsp8 cofactors included, but also RNA double strands [20,21,22,27]. In our work, we want to address a proposal for a reasonable inhibition mechanism for Penciclovir, still unknown [28], describing not only features of the non-covalently bound drug within the active site, but also in the bound pre- and post-translocation states. To better interpret the results, a comparison with data from MD simulations carried out in the presence of a natural base, guanosine triphosphate (GTP), was performed.

Furthermore, importantly, this study opens the way for MD approaches to determine, in silico, possible mechanisms of action for other NA drugs.

## 2. Results and Discussion

In this paper, structural, dynamical, and mechanism insights were obtained regarding the action of Penciclovir towards RdRp of SARS-CoV-2.

As described in the Section 1, for the sake of completeness and because of the crucial roles of the cofactors in the replication complex, MD simulations were performed on a much larger system containing not only the nsp12 protein, but also the cofactors nsp7, nsp8a, and nsp8b (see Figure 2), known for stabilizing the double-strand RNA during the replication process. The charged lysine side chains of the nsp8a and nsp8b helices point inside the tunnel containing the growing dsRNA and stabilize the system by means of electrostatic interaction with negative phosphate groups of the dsRNA backbone [3,28].

In order to study how the drug may influence the various steps of the RNA elongation process, with the aim of identifying the key features of the RNA duplication inhibition mechanism by PENT, the major differences between the replication complex with the drug PENT and the native binding partner GTP during this process were analyzed.

In this context, indeed, in the literature, in the unbound state or bonded in the +1 or -1 position, various residues of protein complex in the active site can interact with the drug/base (i.e., N691, R555, R551, R553, D623, S682) by means of hydrogen bonds and electrostatic interactions determining the overall stability of the complex [2,3,8,10,29]. Moreover, it should be noted that specific interactions of base/amino acid active site can, in turn, provoke changes in the distance/orientation of protein groups not directly involved in this interaction, but fundamental, for example, in the catalytic action from unbound to bound state or in determining the accessibility of the active site for an incoming NTP. For example, the coordination of the bivalent ions by the COO- group of D618 as well as distances of MA with Pα and Y619 and MB with O3′ of U23 are fundamental in the feasibility of the catalytic event [10,30]. Moreover, the mobility of R555, R553, and R551 and of the bivalent ions as well as the absence of coordination of COO- of D618 with MA or MB are indicative of an active-site-opened state, accessible to the entrance of another NTP [3,29,30].

**Figure 3:**
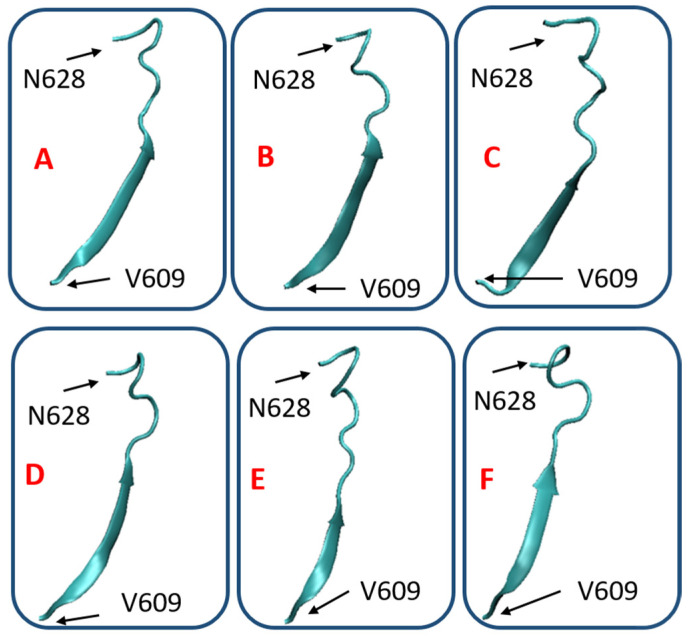
Structural variations of motif A of nsp12 (region: V609-N628) in nbGTP (**A**), G24 (**B**), G23 (**C**), nbPENT (**D**), PEN24 (**E**), and PEN23 (**F**) complexes.

In this work, the starting point was the APO structure, that is, the protein/dsRNA complex without any drug inside, obtained after MD simulation. Several configurations of proteins/dsRNA/drug complex were simulated (see also Table S1 for acronyms used below) and reported in Section 2.1, Section 2.2, and Section 2.3: (1) with PENT or GTP in the active site in correspondence with the +1 position of the product RNA strand but in unbound state (called nbPENT and nbGTP, respectively); (2) with drug or base (here in the monophosphate form and thus called PENC and GUA) covalently linked to the product RNA strand in the active site in correspondence with the +1 position (called PEN24 or G24); (3) with PENC or GUA covalently linked to the product RNA in correspondence with the -1 position (called PEN23 and G23). It should be noted that, according to DSSP data (results not shown), the complex shows, overall, apparently few different features if the synthetic drug or the natural base are present in the active site in all the three configurations analyzed; major variation between complexes with drug or base are, for example, in the motif A (amino acids region: V609-N628) as reported in Figure 3, in accordance with the literature [2]. This result indicates that the entrance, binding, and translocation of the base or drug does not influence the overall features of the complex, and major variations occur in the active site, as explained earlier.

On the basis of these results, to evaluate the differences between the simulations, the interactions of GTP (or GUA) or PENT (or PENC) in the complex with amino acids present in the active site, with the adjacent base in the product strand and with the complementary base in the template strand are analyzed and presented in the following. Moreover, changes are also monitored in the distance of protein groups not directly involved in the interaction of the drug (or base) and the protein/dsRNA complex, which causes key variation in the overall asset of the protein complex (i.e., the coordination of an incoming NTP or drug).

### 2.1. MD Simulations of GTP or PENT in the Active Site (Unbound State)

**Figure 4:**
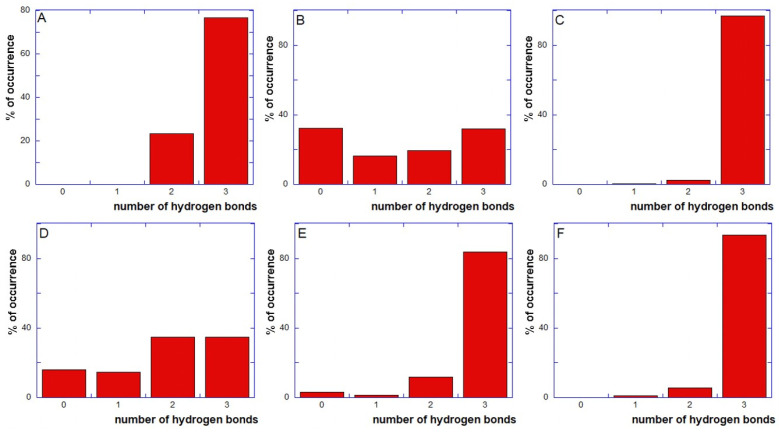
Percentage of occurrence of one, two, or three hydrogen bonds with the complementary base of (**A**): GTP; (**B**): PENT; (**C**): G24; (**D**): PEN24; (**E**): G23; and (**F**): PEN23. Standard deviation is below 5%.

**Figure 5:**
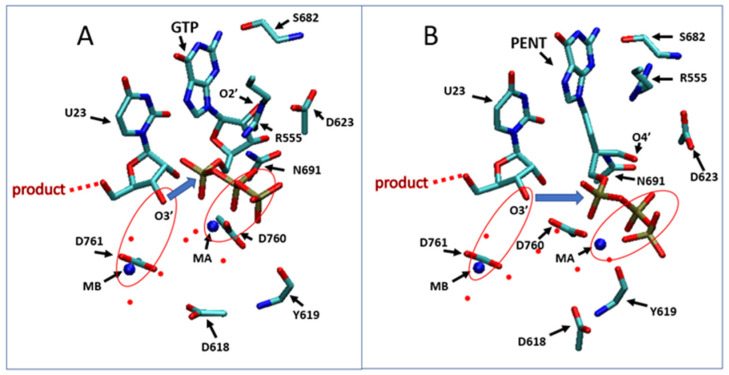
Images of active site of the complex in the presence of (**A**): GTP or (**B**): PENT. For clarity, except for S682 and Y619, only the side chains of amino acids are shown. Red bullets are the oxygen of water molecules surrounding ions. Images have been obtained by VMD software.

Taking these in mind, simulations show that GTP (unbound state) is located in the active site, firmly stabilized by a complex network of hydrogen bonds, in accordance with the literature [2,3,8,31]. As shown in Figure 4A, GTP is indeed involved always in at least two, but more frequently three hydrogen bonds with the complementary base of the template strand. This interaction is a first necessary step in the stabilization process [3]. Moreover, (Figure 5A), hydrogen bonds are also within O2′ and O3′ ribose hydroxyl groups and side chains of D623, N691, and within the CO of the backbone of S682 and N2 (see Scheme 1) of the guanine moiety. Furthermore, the oxygen atom in the phosphate moiety Pα also interacts with the R555 amine side chain (its distance is less than 3.0 Å for the last 50 ns of the simulation time), which is, in turn, stabilized by hydrogen bonds with the side chain of D623. This feature leads the ribose and triphosphate of NTP, as well as the active site, in an arrangement able to facilitate the subsequent binding process [10,31]. As shown in Figure 5A, MA is in proximity of the Pα, Pβ, and Pγ moieties, thus assisting in the exit of the pyrophosphate group, while MB is close to D761 carboxylate and the O3′ of U23, thus lowering its pKa. D618, moreover, is nearby both the two ions, MA and MB.

In the case of PENT, instead the drug is inserted in the active site but is not firmly stabilized by hydrogen bond networks (HBs) because it lacks of the ribose moiety, thus lacking the ability to form these bonds with D623 and N691, as shown in Figure 5B. HBs between O4′ of PENT and the amine side chains of R555, as well as between N2 of the guanine moiety and the hydroxyl group of S682, may be formed (the distance among groups are below 3.0 Å for 40% and 34% of the total time under investigation, respectively). Furthermore, PENT is not stabilized by HBs with the complementary base as much as GTP: it can spend about 30% of the total time without forming any HBs, and for only about 50% of the total time, it is able to form two or three HBs with the complementary base (Figure 4B).

Nevertheless, interestingly, in this case, the arrangement of the ions and residues involved in the binding event is similar to that obtained in the case of nbGTP (Figure 5B). By comparing results obtained with GTP and PENT present in the active site, it is possible to conclude that PENT can insert into the active site, even if is not stabilized by a relatively high number of interactions (on the contrary GTP, as explained above, is well stabilized in the active site), and the binding of PENT to the product strand is plausible and cannot be discarded. This implies, in turn, that the inhibition activity of PENT may not be exerted at this stage.

### 2.2. MD Simulations of Guanine (GUA) or Penciclovir (PENC) Linked to the Product RNA Strand in the Active Site in the +1 Position

As reported in Section 2.1, PENT can enter the active site and be coordinated by amino acid residues present in it, even if to a lesser extent than GTP. We have therefore investigated features of the complex once the base or drug is linked to the product RNA strand, thus performing a comparison of the G24 e PEN24 systems.

**Figure 6:**
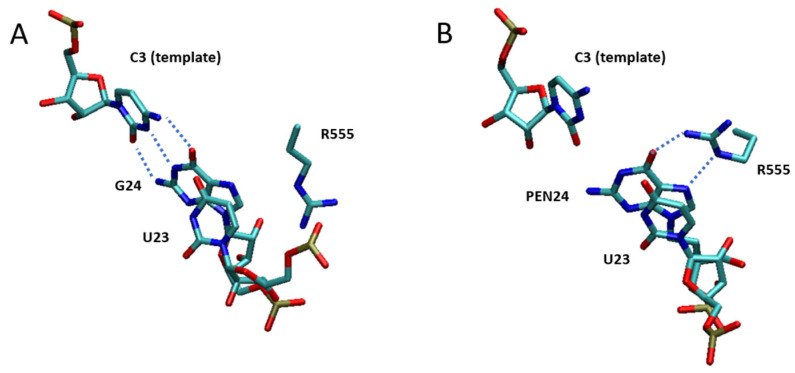
Images of the C3 (see Figure 1) in the template strand and side chain of R555 in (**A**): G24 and (**B**): PEN24. For clarity, hydrogen bonds are depicted by dots. Images were obtained by VMD software.

As reported above, in the unbound state, GTP is stabilized by a huge number of interactions; this is not the case for G24, as shown in Figure 6A, which instead exhibits a high flexibility in the active site. O2′ and O3′ of GUA, indeed, do not form HBs with the carboxylate group of D623 (average distance is 5.7 ± 1.1 Å) and NH2 of N691 (distance is always more than 10 Å). Furthermore, the great distance variation and the lack of HBs between Nη of R555 and the carboxylate group of D623 (average distance 5.2 ± 2.4 Å) suggest flexibility of the active site itself. It should be borne in mind that once bound, the base/drug must translocate; in this context, therefore, the presence of HBs with amino acids of the active site, able to stabilize them, disadvantages dsRNA movement. Interestingly, GUA forms, for almost the full duration of the simulation, three HBs (Figure 4C) with the complementary base, thus indicating a strong interaction between the two. All of these features are in line with the feasibility of the translocation process, which implies a concerted movement of the dsRNA in the enzymatic complex. On the contrary, PENC in PEN24 is strongly stabilized in the active site by two HBs (Figure 6B); one is between the Nε of R555 and N7 of PENC and the other with Nη of R555 and O6 of PENC (these HBs are formed for 96% of the total time; see Figure S3). It should be noted that PENC is not able to establish stable HBs with the complementary base (Figure 4D). These results therefore strongly suggest an inhibition or slowing down of the translocation event.

Summarizing, whilst GUA in the bounded form in the +1 position shows a lack of interaction with amino acids of the active site and at the same time is strongly linked to the template stand by three HBs, this is not the case for PENC. In the first case, therefore, translocation may occur easily, while, for PEN24, a weak interaction with the complementary base of the product strand and strong interactions with amino acids in the active site represent disfavorable factors for translocation. It is plausible, therefore, that inhibition could occur at this stage.

### 2.3. MD Simulations of Guanine (GUA) or Penciclovir (PENC) Linked to the Product RNA Strand in the Active Site and in the -1 Position

**Figure 7:**
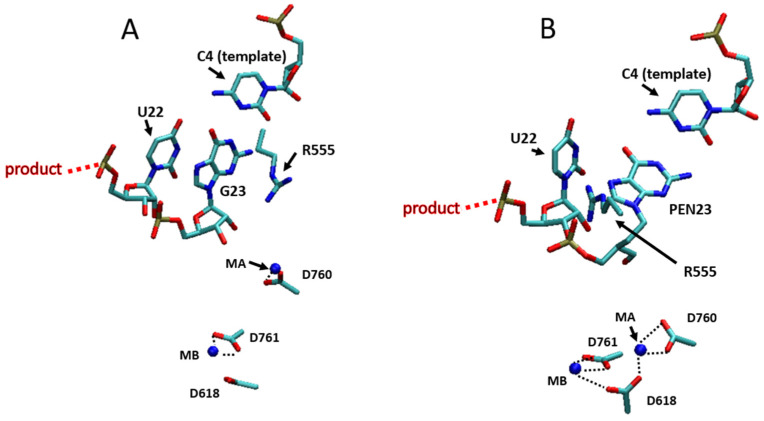
Images of the bases in the template and product strands, ions, and side chain of some amino acids in the active site in the presence of G23 (**A**) and PEN23 (**B**). Images were obtained by VMD software.

Notwithstanding results reported in the Section 2.2 showing that in the presence of drug covalently linked to the product strand, translocation could be much more difficult than for GUA, we have characterized the features of the systems in which the GUA or PENC are in the -1 position (PEN23 or G23). A comparison between G23 and PEN23 features (Figure 7) allows us to determine if the activity of PENC takes place also at this point in inhibiting or slowing down the replication process.

First of all, in the G23 complex (Figure 7A), D618 does not coordinate the MA ion; both MA and MB ions are far apart and mobile, as suggested by the obtained average distance and high standard deviation (distance = 8.3 ± 0.8 Å). At the same time, R555 is also very mobile (the distance between Pα of PENC and C_ζ_ of R555 is 10.3 ± 2.4 Å), far apart from G23, and thus able to coordinate an incoming NTP. All of these data indicate their availability to accommodate an incoming NTP.

Finally, as in the G24 state, GUA is still strongly involved in HBs with the complementary base in the template strand (the number of HBs is indeed three for 80% of the time, as shown in Figure 4E), confirming that GUA in position 23 does not destabilize the growing dsRNA.

On the contrary, as shown in Figure 7B, in PEN23, D618 coordinates both the two metal ions. This situation leads to ions being close together with low mobility (distance: 5.6 ± 0.2 Å). Furthermore, in PEN23, R555 is close to the phosphate group of PENC, with its Nη pointing toward the oxygen atoms of the PENC phosphate moieties (distance between Pα of PENC and C_ζ_ of R555 is 6.6 ± 1.6Å), and thus is not more able to exercise its role, which is the interaction with the triphosphate group of a new incoming NTP into the active site. This means that Penciclovir bound after translocation can act as a blocker for the two metal ions and R555, which are important features for the addition of the nucleotides in the product strand [11]. Differences in mobility have been also confirmed, for example, by the root-mean-square-fluctuations (RMSFs) of C_α_ in motif A residues (motif A contains D618 and D623 amino acids), reported in Figure S4A. RMSFs are indeed lower in PEN23 with respect to GUA23. For comparison, RMSF values for the C_α_ in motif A residues in PENs24 and G23 are also reported (Figure S4B); in this last case, values are comparable. This finding also suggests the key role of motif A for the entrance of incoming NTP.

Summarizing, after translocation, if inhibition does not occur, the active site is empty and prone to the entrance of a new NTP; to this end, mobility of R555 and of the bivalent ions as well as the absence of coordination of COO- of D618 with MA or MB are necessary [3,29,30]. These features occur in the G23, but not in PEN23 as, for example, R555 is close to PENC, and the coordination of D618 to both MA and MB is indicative of an active-site-closed state, not more accessible to the entrance of another NTP [3,29,30].

The reason for all of the differences between G23 and PEN23 can be addressed by the absence of the ribose ring in the drug, a major flexibility of the O4’ coming from an aliphatic region of the molecule, and the absence of ring tension that an O3′ might have in the case of a nucleotide. It is interesting to note that like GUA, PENC in the -1 position also forms stable HBs with the complementary base in the template strand (see Figure 4F) and therefore does not destabilize the dsRNA.

## 3. Materials and Methods

All MD simulations were performed using the software Gromacs19 with the version of Force-Field Charmm36 implemented in February 2021 (see below). In detail, the molecular system was inserted into a periodic cubic box and solvated with a suitable number of water molecules (see Table S2 for details). The water molecules were modelled by the simple point charge extended (SPC/E). A first 500 ps equilibration with a timestep of 0.5 fs at 50 K was followed by a 1 ns long annealing from 50 K to 300 K with a time step of 2 fs. A 130 ns production run with a time step of 2 fs was performed for two replicas on each molecular system. Temperature and isotropic pressure were controlled with the velocity-rescale [32] algorithm (τ = 0.6 ps) and with the Berendsen [33] algorithm (τ = 1.0 ps and compressibility = 4.5⋅10^−5^ bar^−1^), respectively, resulting in an NPT ensemble. Long range Lennard-Jones interactions were treated with a cut-off of length 1.4 nm, and electrostatic interactions were calculated with the Particle Mesh Ewald algorithm with the same cut-off [34]. Data reported are taken by the last 100 ns of MD. As shown, for example, in Figure S1 (Supplementary Materials), indeed, reporting the root-mean-square-deviation (RMSD) for motif A when the drug or base is linked to the product strand in -1 position (see below), 30 ns are necessary for the system to rearrange itself, thus obtaining a reliable starting point.

In order to obtain the initial configuration of the whole complex, a merge of three different crystallographic systems has been performed using the software UCSF Chimera [35]. These systems are: (1) pdb id 7BV2 [36] containing SARS-CoV-2 nsp12,7,8a (incomplete), a short dsRNA, and the drug Remdesivir covalently bound to the dsRNA; (2) pdb_id 5F81 containing RdRp, a long dsRNA (taken from EV71), and a CTP entering the active site (which correspond to the intermediate stage) [37]; (3) pdb_id 6YYT containing SARS-CoV-2 nsp12, nsp7, nsp8a, nsp8b and a larger dsRNA (Figure 2) [30]. Three different crystallographic structures have been used because the first one contains the correct sequence of amino acids of nsp12 SARS-CoV-2, the second one contains the GTP not covalently bound on the active site, and the third structure contains nsp8a,b and nsp7 with the longer dsRNA sequence.

The PENT molecule was then built from scratch based on the analog. GTP was superimposed on the CTP of the EV71 X-ray structure using the software UCSF Chimera. The nucleotide sequence from the larger dsRNA (Figure 1 and Figure 2) was unaltered except for the transformation at the -1 position of the adenine in the template with a cytosine, when guanine is its complementary base in the product strand. The two Mn^2+^ ions were initially positioned in accordance with those found in the EV71 crystal structure. Preliminary simulations of dsRNA in water and into the protein complex were performed with the Gromos54a7 force-field to ensure that the dsRNA would remain in its double-stranded conformation with the base pair coupling. However, as shown in Figure S2, in a few ns the double strand loses its close conformation. These results suggest that Gromos54a7 is not able to reproduce the correct combination of hydrogen bonds driving the correct orientations of the base pairs of the dsRNA in any environment. To overcome this drawback, the most recent version of Charmm36 force-field (modified ad hoc for the nucleic bases in February 2021) was used [38,39]; in this way, the double-strand RNA remains in its stable conformation during all the simulations both in water and in the enzyme.

The persistence time of a hydrogen bond was estimated by multiplying by the time step (100 ps) the number of times in which the distance between the pair of atoms considered was between 2.5 and 3.0 Å (typical interval of the hydrogen bond). The percentage was obtained from the ratio between this persistence time and the total simulation time. Images were obtained by VMD [40].

## 4. Conclusions

In this article, we indicate at which stage of the replication process Penciclovir exerts its inhibition action against RdRp of SARS-CoV-2 by MD simulation. The importance of this article resides not only in this result, but also in the fact that, to obtain this, for the first time to the best of our knowledge, MD simulations have been performed including a stable ds-RNA and, as regards the RdRp complex, not only nsp12, but also the nsp7 and nsp8 cofactors. Furthermore, not only we have characterized the non-covalently bound drug within the active site, but also in the bound pre- and post-translocation state. MD simulations indeed indicate that the drug, similar to the natural base, can enter into the active site and bind to the product strand. This indicates that Penciclovir does not inhibit these stages. On the contrary, after bonding to the product strand, a strong stabilization of the drug in the active site can disfavor the translocation, differently to Guanine. This feature suggests that this is the main cause of inhibition. Moreover, MD simulations performed when PENC or GUA is in the -1 position indicate that, in the presence of the drug, unlike GUA, the active site remains in an arrangement unfavorable to the entrance of an incoming NTP, thus strengthening the PENC inhibition. Finally, at the same time, these results open the way for up-to-date MD simulations to determine possible mechanisms of action for other NA drugs.

## Data Availability

Not applicable.

## Supplementary Materials

The following supporting information can be downloaded at: https://www.mdpi.com/article/10.3390/molecules28010191/s1, Further information on RdRp: Table S1 list of acronyms, with figure and full names of drug and base used in molecular dynamic simulations of systems under investigation; Figure S1. Root-mean-square-deviation (RMSD) for motif A when Penciclovir or Guanine are linked to the product strand and are in −1 position; Figure S2: dsRNA in the protein complex, after simulation with GROMOS-54-a7 force-field. Table S2: number of water molecules used in the simulation box for each system under study; Figure S3. Percentage of occurrence of one or two hydrogen bonds (HBs) between Nε of R555 and N7 of PENC and Nη of R555 and O6 of PENC in PEN24; Figure S4. root-mean-square-fluctuactions (RMSF) of Cα in motif A residues in G23, PEN23, G24 and Pen24; Video S1: D618, D760 and D761 as well as R555 and Guanine in G23; Video S2: D618, D760 and D761 as well as R555 and PENC in PEN23 [1,2,3,14,29,41].
