# Supplementary material for: Inspection on the Mechanism of SARS-CoV-2 Inhibition by Penciclovir: A Molecular Dynamic Study
Source: Molecules. 2022 Dec 26;28(1):191. doi: 10.3390/molecules28010191 (PMC9821970; doi:10.3390/molecules28010191)
Supplement: Supplementary file 1 [file molecules-28-00191-s001.zip › molecules-2097363-supplementary.pdf]

molecules

SUPPLEMENTARY MATERIALS

Inspection on the Mechanism of SARS-CoV-2 Inhibition by Penciclovir: a Molecular Dynamic Study

Micaela Giannetti , Claudia Mazzuca *, Giorgio Ripani and Antonio Palleschi *

Department of Chemical Science and Technologies, University of Rome "Tor Vergata", Via della Ricerca Scientifica, 00133 Rome, Italy
* Correspondence: claudia.mazzuca@uniroma2.it (C.M.); antonio.palleschi@uniroma2.it (A.P.)

1. Introduction

The RdRp protein

As reported in the main manuscript, the core structure of the RdRp [3,14] divided in three domains [1], fingers, palm and thumb ones, that are are in the regions: V398-A581 and N628-T687; H816-H932; T582-P627 and A688-Q815, respectively. The whole nsp12 comprehends a Nidovirus RdRp Associated Nucleotidyl transferase domain (NiRAN), an Interface and the RdRp domain. Furthermore,nsp12 RNA polymerase exerts its function with the help of several cofactors. More in detail, the nsp7-nsp8 heterodimer binds above the thumb subdomain and stabilizes the thumb-finger interface. Nsp7 makes a major contribution to the binding of the heterodimer to nsp12, while nsp8 only contacts a few residues from nsp12. The other copy of nsp8 (nsp8-2) sits atop the finger subdomain and forms additional interactions with the interaction subdomain. In this structure, similar to other positive-strand RNA RdRp, the template/product entry channel, NTP entry channel, and nascent strand exit channel are all positively charged and converge in a central cavity, which is the active site of the SARS-CoV-2 RdRp [14]. In this active site, these RdRp motifs mediate template-guided RNA synthesis. Motif A (V609-N628) houses the catalytic motif DX2-4D, in which the first aspartate D618 is invariant in most viral polymerases. The flexible loop in Motif B (T680-T710) serves as a hinge to undergo conformational arrangement associated with template RNA and substrate binding. Motif C (F753-N767) contains the catalytic motif that is D760 and D761, which is essential for binding the metal ions. Motif D contains residues L775-E796. Motif E contains residues H810-V820 and combines with the palm subdomain to support the template chain. Motif F (L544-V560) interacts with the phosphate group of incoming NTP. The NTP entry channel is formed by a set of charged residues, including K545, R553 and R555 in motif F [41]. Motif G (K500-S518) interacts with the template strand [2,3,39].

2. Results and Discussion

Table S1. Acronyms of drug and base used in molecular dynamic simulations of systems under investigation.

<table>
<tr><td>nbGTP
Unbound Guanine-triphosphate in the active site near the +1 position of the product RNA strand</td><td>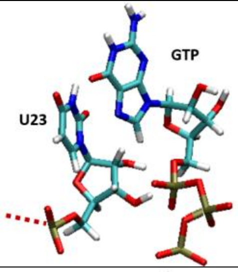
</td></tr>
<tr><td>nbPENT
Unbound Penciclovir-triphosphate near the +1 position of the product RNA strand</td><td>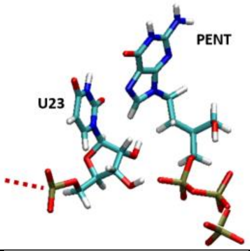
</td></tr>
<tr><td>G24
Guanine-monophosphate covalently linked to the product RNA strand in the +1 position</td><td>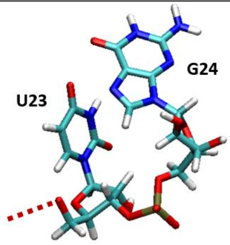
</td></tr>
<tr><td>PEN24
Penciclovir-monophosphate covalently linked to the product RNA in the +1 position</td><td>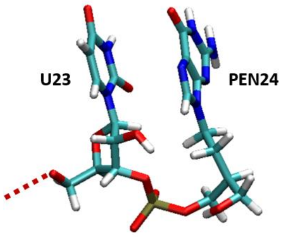
</td></tr>
<tr><td>G23
Guanine-monophosphate covalently linked to the product RNA strand in the -1 position</td><td>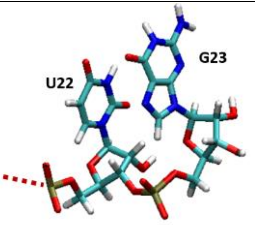
</td></tr>
<tr><td>PEN23
Penciclovir-monophosphate covalently linked to the product RNA strand in the -1 position</td><td>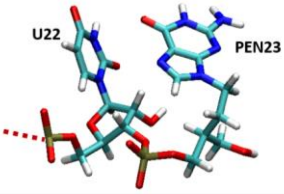
</td></tr>
</table>

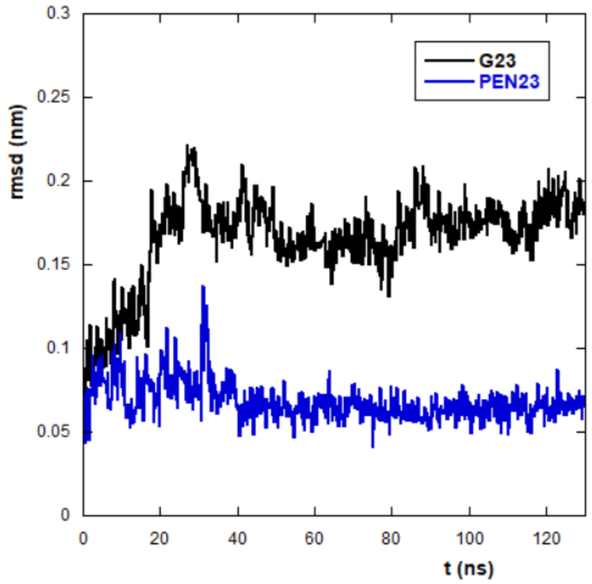

Figure S1. Root-mean-square-deviation (RMSD) for motif A when Penciclovir (blue, PEN23) or Guanine (black, G23) are linked to the product strand and are in -1 position.

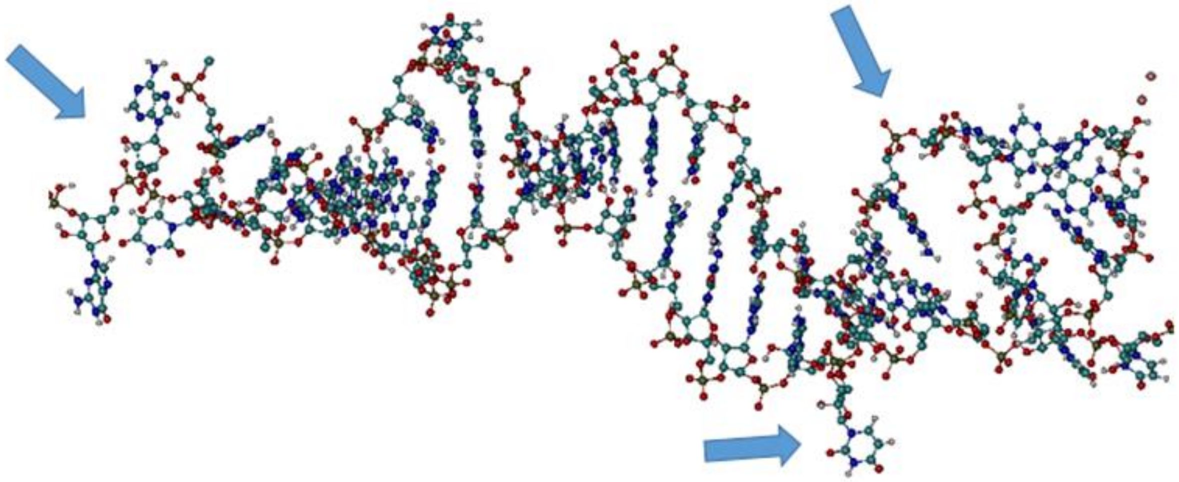

Figure S2. dsRNA at the end of the simulation using the GROMOS-54-a7 force field. Arrows indicate the loose of base pairing and opening of the double strand.

Table S2. Dimensions of the box (in nm) and number of water molecules (nW) used in the MD simulations.

	Box (nm × nm × nm)	nW
GTP	15.45979 × 15.45979 × 15.45979	114906
PENT	15.45979 × 15.45979 × 15.45979	114907
G24	15.45979 × 15.45979 × 15.45979	114818
PEN24	15.45979 × 15.45979 × 15.45979	114750
G23	15.45979 × 15.45979 × 15.45979	114871
PEN23	15.45979 × 15.45979 × 15.45979	118370

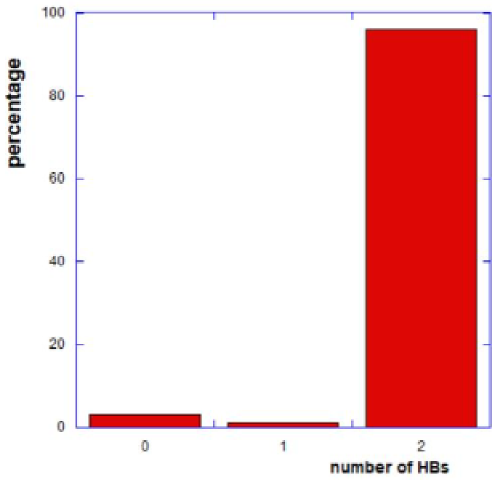

Figure S3. Percentage of occurrence of one or two hydrogen bonds (HBs) between Nε of R555 and N7 of PENC and Nη of R555 and O6 of PENC in PEN24. .

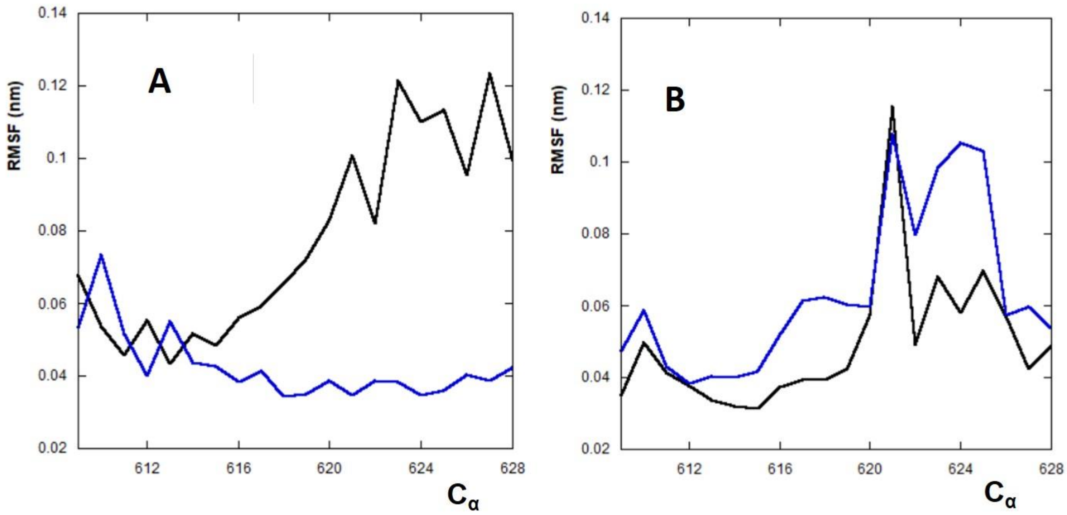

Figure S4. root-mean-square-fluctuactions (RMSF) of Cα in motif A residues in (**A**): G23 (black) and PEN23 (blue); (**B**): G24 (black) and Pen24 (blue**).**

Video S1. D618, D760 and D761 as well as R555 and Guanine in G23
https://drive.google.com/file/d/13NuJh22Ft2ZX2vusvdgO4bEld1sFNsXn/view?usp=share_link

Video S2. D618, D760 and D761 as well as R555 and PENC in PEN23.
https://drive.google.com/file/d/1UQu2i7WLmqg8KOYzWcHo6sW8Jsp7-opF/view?usp=share_link
